# Development and validation of a novel risk assessment model to estimate the probability of pulmonary embolism in postoperative patients

**DOI:** 10.1038/s41598-021-97638-0

**Published:** 2021-09-10

**Authors:** Mao-feng Wang, Fei-xiang Li, Lan-fang Feng, Chao-nan Zhu, Shuang-yan Fang, Cai-min Su, Qiong-fang Yang, Qiao-ying Ji, Wei-min Li

**Affiliations:** 1 Department of Biomedical Sciences Laboratory, Affiliated Dongyang Hospital of Wenzhou Medical University, Dongyang, 322100 Zhejiang China; 2 Department of Cardiology, Affiliated Dongyang Hospital of Wenzhou Medical University, Wuning West Road No. 60, Dongyang, 322100 Zhejiang China; 3 Department of Respiratory, Affiliated Dongyang Hospital of Wenzhou Medical University, Dongyang, 322100 Zhejiang China; 4 Shanghai Key Laboratory of Artificial Intelligence for Medical Image and Knowledge Graph, Hangzhou, 310000 Zhejiang China

**Keywords:** Cardiology, Risk factors, Signs and symptoms

## Abstract

Pulmonary embolism (PE) is a leading cause of mortality in postoperative patients. Numerous PE prevention clinical practice guidelines are available but not consistently implemented. This study aimed to develop and validate a novel risk assessment model to assess the risk of PE in postoperative patients. Patients who underwent Grade IV surgery between September 2012 and January 2020 (n = 26,536) at the Affiliated Dongyang Hospital of Wenzhou Medical University were enrolled in our study. PE was confirmed by an identified filling defect in the pulmonary artery system in CT pulmonary angiography. The PE incidence was evaluated before discharge. All preoperative data containing clinical and laboratory variables were extracted for each participant. A novel risk assessment model (RAM) for PE was developed with multivariate regression analysis. The discrimination ability of the RAM was evaluated by the area under the receiver operating characteristic curve, and model calibration was assessed by the Hosmer–Lemeshow statistic. We included 53 clinical and laboratory variables in this study. Among them, 296 postoperative patients developed PE before discharge, and the incidence rate was 1.04%. The distribution of variables between the training group and the validation group was balanced. After using multivariate stepwise regression, only variable age (OR 1.070 [1.054–1.087], *P < *0.001), drinking (OR 0.477 [0.304–0.749], *P* = 0.001), malignant tumor (OR 2.552 [1.745–3.731], *P* < 0.001), anticoagulant (OR 3.719 [2.281–6.062], *P* < 0.001), lymphocyte percentage (OR 2.773 [2.342–3.285], *P* < 0.001), neutrophil percentage (OR 10.703 [8.337–13.739], *P* < 0.001), red blood cell (OR 1.872 [1.384–2.532], *P* < 0.001), total bilirubin (OR 1.038 [1.012–1.064], *P* < 0.001), direct bilirubin (OR 0.850 [0.779–0.928], *P* < 0.001), prothrombin time (OR 0.768 [0.636–0.926], *P* < 0.001) and fibrinogen (OR 0.772 [0.651–0.915], *P* < 0.001) were selected and significantly associated with PE. The final model included four variables: neutrophil percentage, age, malignant tumor and lymphocyte percentage. The AUC of the model was 0.949 (95% CI 0.932–0.966). The risk prediction model still showed good calibration, with reasonable agreement between the observed and predicted PE outcomes in the validation set (AUC 0.958). The information on sensitivity, specificity and predictive values according to cutoff points of the score in the training set suggested a threshold of 0.012 as the optimal cutoff value to define high-risk individuals. We developed a new approach to select hazard factors for PE in postoperative patients. This tool provided a consistent, accurate, and effective method for risk assessment. This finding may help decision-makers weigh the risk of PE and appropriately select PE prevention strategies.

## Introduction

Pulmonary embolism (PE) is a serious life-threatening disease with potentially fatal outcomes and represents a serious public health problem affecting 350 thousand to 600 thousand Americans annually^1,2^. The risk of PE is high after surgery, while PE remains the most preventable cause of death in hospitalized patients. PE is known to cause significant morbidity with associated health-care costs. Timely and accurate diagnosis, reasonable risk stratification and strict anticoagulant therapy are the keys to the prognosis of PE^3,4^ and have been shown to reduce mortality^5,6^. However, PE in postoperative patients remains largely underdiagnosed due to nonspecific clinical manifestations that are easily overlooked by inexperienced eyes^7–9^. In addition, these conditions have a negative impact on the quality of life and increase the burden on patients and the whole medical system.

PE is one of the fatal complications of postoperative patients. It is difficult to understand the mechanism of embolism within a few hours to a few days^10^. Before the operation, surgeons should thoroughly examine the patient to determine the risk of embolism. A comprehensive understanding of those patients and assigning them to appropriate risk categories will effectively prevent the incidence of postoperative embolism^11^. With these concerns, a number of studies have focused on the risk factors and incidence of PE in postoperative patients. The reported event rate of PE varied from 0.25 to 4.6% when prophylaxis was used^12^. For different postoperative patients, timely and correct diagnosis at the right time is the key to better prognosis. Despite the continuous development of technology and research, there are still some difficulties in the diagnosis of PE. Therefore, it is necessary to fully understand the clinical manifestations of patients and use appropriate diagnostic methods and prediction models to make timely diagnoses. Due to the emphasis on joint decision-making, predictive tools in surgery are becoming increasingly important. To date, there are various risk assessment models (RAMs) for PE, including the Caprini RAM^13^ and Rogers RAM^14^.

To date, it remains unclear which RAM should be routinely used to identify at-risk patients for PE. Existing RAMs to evaluate the risk of PE in acutely ill medical patients are difficult to compare, and none fulfills the criteria of an ideal RAM^15^. Therefore, the risk assessment of PE may be improved by developing and validating a simple RAM.

Thus, in this study, we used a large sample of postoperative patients in a comprehensive hospital in China to develop and validate a simple RAM to determine the possible risk factors for PE in postoperative patients. This study is helpful for risk stratification and to formulate individual prevention strategies and reasonable PE prophylaxis protocols.

## Methods

### Ethics statement

This retrospective study was approved by the Medical Ethics Committee of Affiliated Dongyang Hospital of Wenzhou Medical University (No: 2019-YX-059), who waived the need for informed consent in the study. All clinical and laboratory variables included in this analysis were retrospectively collected. Patient records/information were anonymized and deidentified prior to analysis. Our research was performed in accordance with the Declaration of Helsinki.

### Participants and sample collection

**Figure 1:**
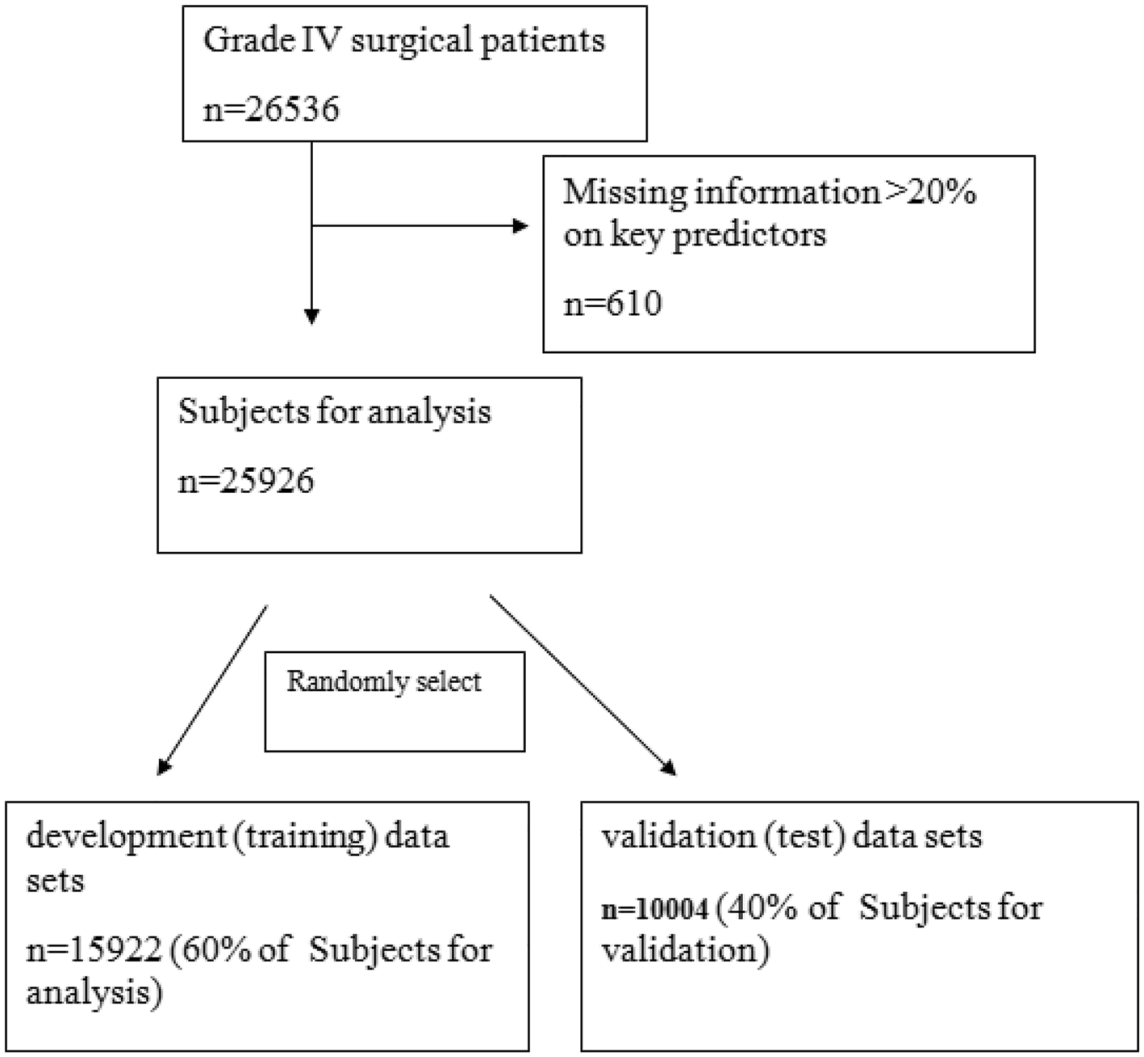
Flow diagram of subjects.

According to the difficulty and complexity of surgery, the health administration of China divided the surgery into four grades (I–IV), in which Grade IV surgery is a major operation with high risk, a complicated process and high technical difficulty. The medical records of 26,536 patients (Fig. 1) who underwent Grade IV surgeries at the Affiliated Dongyang Hospital of Wenzhou Medical University were retrospectively reviewed and analyzed from September 2012 to January 2020. The primary endpoint of the analysis was PE defined according to the criteria of the European Society of Cardiology Guidelines, and PE was confirmed by an identified filling defect in the pulmonary artery system in CT pulmonary angiography (CTPA), including subsegmental PE. The PE incidence was evaluated before discharge. The follow-up period ended upon patient discharge from the hospital. Patients with missing information, especially those who did not undergo PE-related imaging or blood tests before surgery, were excluded. All preoperative data containing clinical and laboratory variables were extracted for each participant.

### Statistical analysis

SAS 9.4 and R i386 4.0.0 software for Windows were used for statistical analysis. Continuous variables were expressed as the mean ± standard deviation (SD) or medians and interquartile range (IQR) and were compared by either Student’s *t* test or the Mann–Whitney *U* test. Categorical variables were compared by χ^2^ test or Fisher’s exact test. We split the data into development (training) and validation (test) data sets. Sixty percent of the data were randomly selected as the training set; the validation data included the rest. To ensure the reliability of the data, we excluded patients who had > 20% missing information on key predictors: age, sex, surgical grade, and PE-related imaging or blood tests. Any remaining missing predictor values in the development data were imputed by use of multiple imputation techniques (e.g. expectation maximization approach). Model variable selection was divided into three steps: Step 1: use stepwise regression to select variables that were significantly related to PE and without collinearity; Step 2: use the random forest algorithm to rank the importance of these variables; Step 3: increasing the variables one by one according to the importance from high to low, and only retains the variables that have a large impact on the performance in the final model. Finally, a novel risk assessment model (RAM) for PE was developed with multivariate regression and was evaluated on a validation set. The discriminative ability of the RAM was assessed by the area under the receiver operating characteristic curve (AUC), and model calibration was evaluated by the Hosmer–Lemeshow statistic. The random forest algorithm was used to rank the importance of variables in the model. The null hypothesis was rejected for *P* < 0.05.

## Results

### Characteristics of the study population

A total of 26,536 patients with Grade IV surgery were included in this study. We excluded 610 patients who had > 20% missing information on key predictors, and the remaining 25,926 postoperative patients were recruited for analysis. We split the postoperative patients into development (training) and validation (test) datasets and randomly selected 15,922 of the patients as the training set. The validation data included the remaining 10,004 patients. Figure 1 describes the study participants who underwent Grade IV surgery and the reasons for exclusion. Table 1 presents the basic characteristics of the study population. After excluding 92 variables with missing information more than 20%, we included 53 clinical and laboratory variables of 25,926 patients in this study (see “Appendix 1”). There are missing data for 38 variables with percentage ranged from 2.64 to 18.25%, and expectation maximization approach was used to impute the missing data. Among 25,926 patients, 296 postoperative patients developed PE before discharge, and the incidence rate was 1.04%. The distribution of variables between the training group and the validation group was balanced, and there was no significant difference in the distribution of all analysis factors between the two datasets (Table 1).

**Table 1:** Baseline characteristics of subjects.

Variables	Overall (n = 25,926)	Training set (n = 15,922)	Testing set (n = 10,004)	*P* value
Age (median [IQR])	55.00 [45.00, 67.00]	55.00 [45.00, 67.00]	55.00 [44.00, 67.00]	0.553
Drinking (%)	6363 (24.5)	3898 (24.5)	2465 (24.6)	0.784
Malignant.tumor (%)	7058 (27.2)	4382 (27.5)	2676 (26.7)	0.178
Anticoagulant (%)	13,619 (52.5)	8405 (52.8)	5214 (52.1)	0.299
Lymphocyte.percentage.last (median [IQR])	0.27 [0.19, 0.34]	0.27 [0.19, 0.34]	0.27 [0.19, 0.34]	0.678
Neutrophil.percentage.max (median [IQR])	6.62 [5.83, 7.63]	6.62 [5.83, 7.63]	6.62 [5.82, 7.65]	0.971
Red.blood.cell.last (median [IQR])	4.39 [4.03, 4.75]	4.39 [4.03, 4.74]	4.39 [4.03, 4.75]	0.872
Total.bilirubin.last (median [IQR])	12.00 [9.00, 15.90]	12.00 [9.06, 15.88]	12.00 [9.00, 16.00]	0.514
Direct.bilirubin.last (median [IQR])	3.90 [2.90, 5.40]	3.90 [2.90, 5.39]	3.90 [2.90, 5.40]	0.489
Prothrombin.time.last (median [IQR])	13.00 [12.50, 13.60]	13.00 [12.50, 13.60]	13.00 [12.50, 13.60]	0.562
Fibrinogen.last (median [IQR])	3.28 [2.80, 3.90]	3.28 [2.80, 3.90]	3.28 [2.80, 3.90]	0.913
Label (%)	269 (1.0)	164 (1.0)	105 (1.0)	0.93

Last: The last test results before the diagnosis of pulmonary embolism.

Max: The maximum test results before the diagnosis of pulmonary embolism.

### Development of a risk prediction model for PE

**Figure 2:**
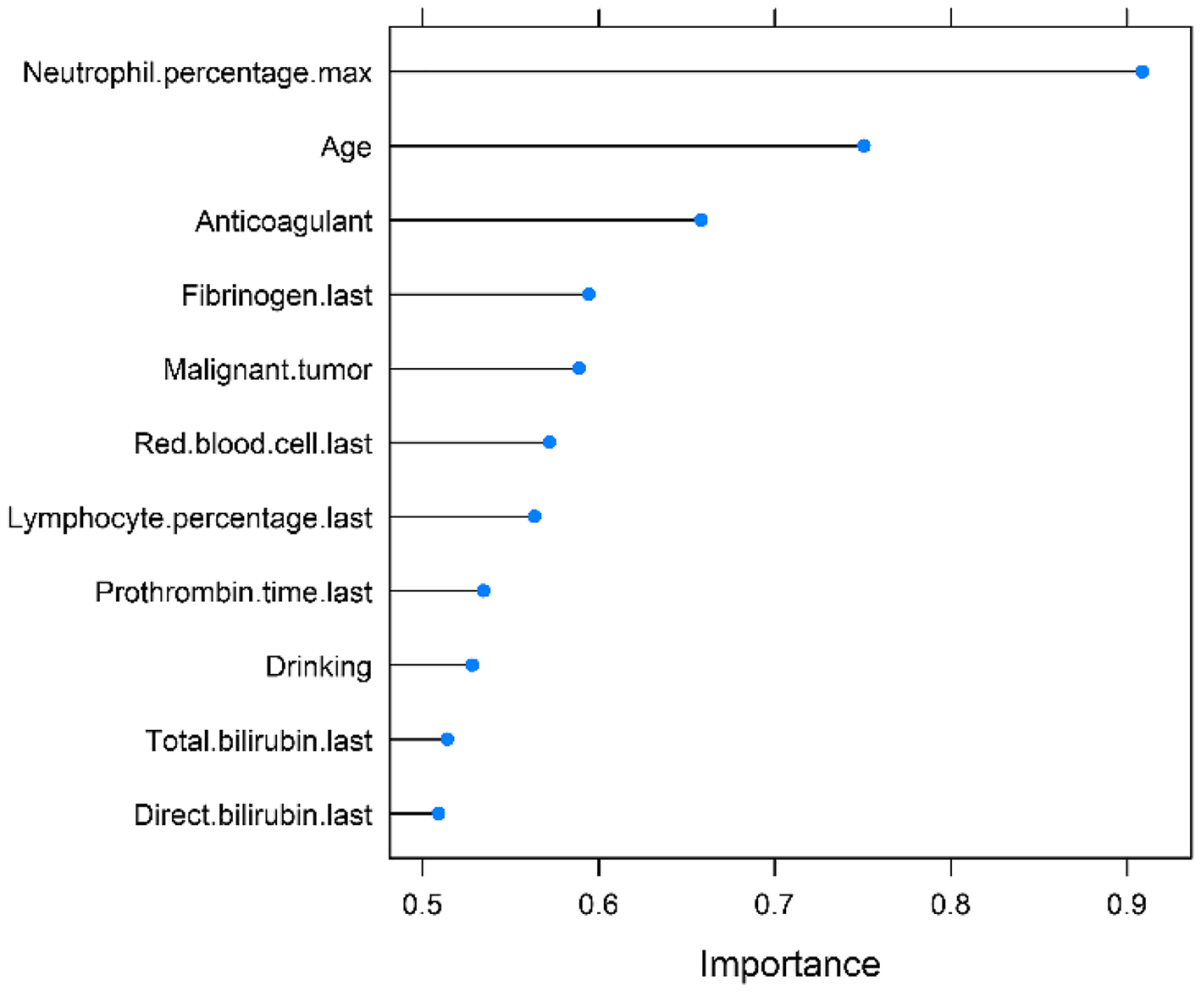
Ranking of the importance of variables in the model. Last: The last test results before the diagnosis of pulmonary embolism. Max: The maximum test results before the diagnosis of pulmonary embolism.

**Figure 3:**
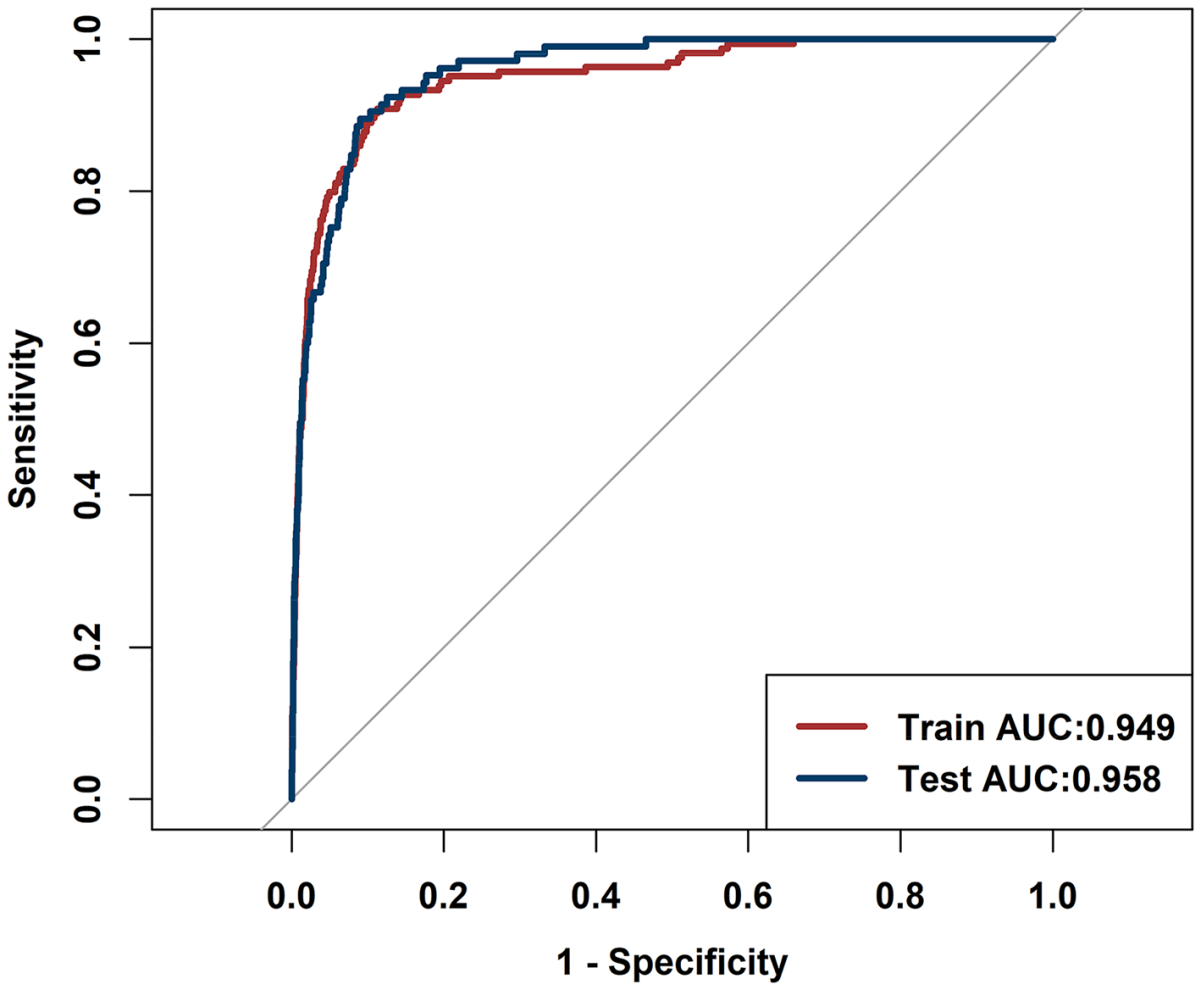
Model ROC curve.

**Figure 4:**
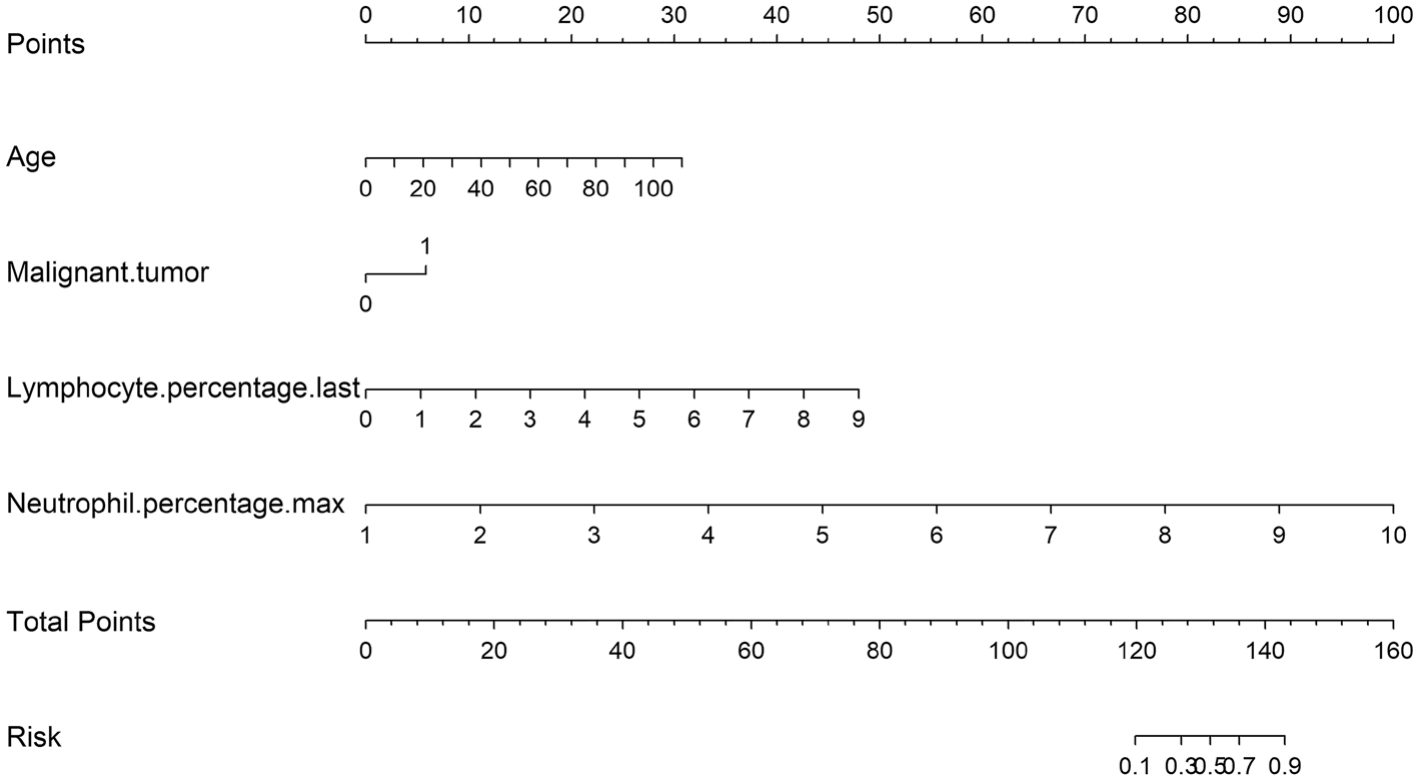
Nomogram curve.

After using multivariate stepwise regression, only variable age (OR 1.070 [1.054–1.087], *P* < 0.001), drinking (OR 0.477 [0.304–0.749], *P* = 0.001), malignant tumor (OR 2.552 [1.745–3.731], *P* < 0.001), anticoagulant (OR 3.719 [2.281–6.062], *P* < 0.001), lymphocyte percentage (OR 2.773 [2.342–3.285], *P* < 0.001), neutrophil percentage (OR 10.703 [8.337–13.739], *P* < 0.001), red blood cell (OR 1.872 [1.384–2.532], *P* < 0.001), total bilirubin (OR 1.038 [1.012–1.064], *P* < 0.001), direct bilirubin (OR 0.850 [0.779–0.928], *P* < 0.001), prothrombin time (OR 0.768 [0.636–0.926], *P* < 0.001) and fibrinogen (OR 0.772 [0.651–0.915], *P* < 0.001) were selected and significantly associated with PE, there was no collinearity between them, except collinearity between total bilirubin and direct bilirubin (see Table 2). The importance of each variable in the model is shown in Fig. 2. The most important variable was the maximum percentage of neutrophils. According to the importance of the variables to the prediction, the variables were added into the model one by one, and the effect of each variable change on the model performance was obtained (see Table 3). The single most influential index reached 0.904. In the end, only the variables that greatly increased the performance of the model were retained, and the final model included four variables, namely, the maximum neutrophil percentage, age, malignant tumor and the last lymphocyte percentage. The AUC of the model was 0.949 (95% CI 0.932–0.966) (Fig. 3), and the *P* value for the Hosmer & Lemeshow test was 0.134. A nomogram to calculate the score and the risk of PE is presented in Fig. 4. The scores of the items displayed in the nomogram should be added up. As it showed in the figure, max neutrophil percentage contributed most to the PE, followed by last lymphocyte percentage, age and malignant tumor. The model can be described with the following equation: probability of PE = e^a^/(1 + e^a^), where a = (− 26.914 + [2.097 * neutrophil percentage] + [0.053 * age] + [1.098 * malignant tumor] + [1.006 * lymphocyte percentage]) (Table 4). Dichotomous variables were classified as equal to 1 for presence and 0 for absence.

**Table 2:** Variables associated with PE.

Variables	Estimate	*P* value	OR [95% CI]	VIF
Intercept	− 28.776	< 0.001		
Age	0.068	< 0.001	1.070 [1.054–1.087]	1.179
Drinking	− 0.740	0.001	0.477 [0.304–0.749]	1.042
Malignant tumor	0.937	< 0.001	2.552 [1.745–3.731]	1.043
Anticoagulant	1.313	< 0.001	3.719 [2.281–6.062]	1.047
Lymphocyte percentage last	1.020	< 0.001	2.773 [2.342–3.285]	4.317
Neutrophil percentage max	2.371	< 0.001	10.703 [8.337–13.739]	4.419
Red blood cell last	0.627	< 0.001	1.872 [1.384–2.532]	1.206
Total bilirubin last	0.037	0.004	1.038 [1.012–1.064]	7.500
Direct bilirubin last	− 0.162	< 0.001	0.850 [0.779–0.928]	7.423
Prothrombin time last	− 0.265	0.006	0.768 [0.636–0.926]	1.093
Fibrinogen last	− 0.259	0.003	0.772 [0.651–0.915]	1.158

Last: The last test results before the diagnosis of pulmonary embolism.

Max: The maximum test results before the diagnosis of pulmonary embolism.

**Table 3:** Model performance according to variables added.

Variables	Model performance	
	AUC	Hosmer and Lemeshow test
Neutrophil percentage max	0.904	0.003
Age	0.92	0.32
Anticoagulant	0.925	0.061
Fibrinogen last	0.924	0.095
Malignant tumor	0.932	0.535
Red blood cell last	0.935	0.082
Lymphocyte percentage last	0.95	0.204
Prothrombin time last	0.951	0.042
Drinking	0.952	0.163
Total bilirubin last	0.952	0.154
Direct bilirubin last	0.95	0.457

Last: The last test results before the diagnosis of pulmonary embolism.

Max: The maximum test results before the diagnosis of pulmonary embolism.

**Table 4:** Final model coefficients.

Variables	Estimate	*P* value
Intercept	− 26.914	< 0.001
Neutrophil percentage max	2.097	< 0.001
Age	0.053	< 0.001
Malignant tumor	1.098	< 0.001
Lymphocyte percentage last	1.006	< 0.001

Last: The last test results before the diagnosis of pulmonary embolism.

Max: The maximum test results before the diagnosis of pulmonary embolism.

### Validation of the risk prediction model

The risk prediction model still showed good calibration, with reasonable agreement between the observed and predicted PE outcomes in the validation set (AUC 0.958). The information on sensitivity, specificity and predictive values according to cutoff points of the score in the training set suggested a threshold of 0.012 as the optimal cutoff value to define high-risk individuals [sensitivity, specificity, positive predictive value (PPV), negative predictive value (NPV) and Youden’s index were 0.909, 0.887, 0.077, 0.999 and 0.796, respectively] (Table 5). The cutoff of 0.012 points was chosen based on the best combination of sensitivity and specificity, with the goal of identifying high-risk patients as well as avoiding PE.

**Table 5:** Model evaluation.

Index	Training set	Testing set
AUC	0.949 [0.932–0.966]	0.958 [0.944–0.972]
ACC	0.888 [0.888–0.888]	0.91 [0.91–0.91]
SEN	0.909 [0.864–0.953]	0.895 [0.837–0.954]
SPE	0.887 [0.882–0.892]	0.91 [0.904–0.915]
PLR	8.07 [7.559–8.616]	9.924 [9.065–10.864]
NLR	0.103 [0.064–0.167]	0.115 [0.066–0.201]
PPV	0.077 [0.066–0.089]	0.095 [0.077–0.114]
NPV	0.999 [0.998–0.999]	0.999 [0.998–1]

AUC: Area under the curve; ACC: Accuracy; SEN: Sensitivity; SPE: Specificity; PLR: Positive likelihood ratio; NLR: Negative likelihood ratio; PPV: Positive predictive value; NPV: Negative predictive value.

## Discussion

This study developed and validated a simple RAM to determine the possible risk factors for PE in postoperative patients. The novel score showed reasonable discrimination and calibration on a large sample of postoperative patients in a comprehensive hospital in China and has the potential to inform decision making on PE. We confirmed the role of well-known risk factors associated with PE, such as age and tumor at baseline. Similarly, covariates that have been previously associated with PE, such as anticoagulant and fibrinogen, remained associated with PE occurrence. Additionally, we featured the relevance of neutrophil percentage, which is commonly appraised in practice and emerged as the strongest predictor of PE in our score. Our final model included four variables: neutrophil percentage, age, malignant tumor and lymphocyte percentage. Two blood cell analysis indexes are important factors, and we think it may be the oxidative stress response caused by acute pulmonary embolism. Previous studies have also confirmed this hypothesis^16–18^.

This new approach enables us to identify the risk factors contained in RAM. This RAM can accurately predict PE events while maintaining a relatively simple, suitable model for clinical work. If an RAM provides inaccurate overestimation or underestimation of future events, it may lead to poor management of patient care and medical resources. On the other hand, if the model is highly predictable but difficult to apply, time-consuming, costly or less relevant, it will not be widely used^19^. Prevention of any disease is better than treatment. Prevention of postoperative PE is a challenging task^20,21^. It is very important to evaluate the risk factors for the patient prior to surgery^22^.

In 2012, a study classified the risk into four levels: low, medium, high and extremely high according to the type of operation (whether minor or major), patient age, previous history of venous thrombosis and type of operation^23^. In a retrospective study, a thorough preoperative examination was very important because it is one of the limitations of drawing conclusions on its results^24^. It is very important to know the risk levels of the appropriate patients for the prevention of these patients. Two large-scale retrospective studies included 8860 patients who were prevented according to the risk factors for venous thrombosis^25,26^. In both studies, their results were statistically significant and supported their protocol for the prevention of postoperative embolism.

We developed an RAM for PE in postoperative patients who was similar but not identical to some widely used RAMs in current practice, such as the IMPROVE (International Medical Prevention Registry on Venous Thromboembolism) VTE RAM^27–29^. Compared with IMPROVE VTE RAM, neutrophil percentage is an additional important risk factor^30^. We identified 50 additional candidate risk factors, 4 of which were considered in our PE RAMs, respiratory failure and heart failure in the MITH VTE RAMs^15,29^, and thrombocytosis and leukocytosis in the MITH RAM^4,31^.

Professional associations and institutions have developed a number of PE RAMs. PE is common in postoperative patients because the operation usually involves many large vessels and is highly invasive^32^. In addition, each existing PE model contains many different risk factors, which require that the assessors are extremely familiar with the patient’s medical history and laboratory examination results and that the assessors dynamically score in a timely fashion when risk factors change^33^.

There were several limitations in our study. The main limitation of our study is the retrospective design. Data collection was based on information available on review of the patient medical records. Second, PE severity was not assessable from the retrospective medical records and including subsegmental PE within our study which may lead to overdiagnosis in some cases. Third, the increased risk of VTE following surgery lasts several weeks. Considering that only PE occurs during hospitalization is a major limitation. Fourth, the aim of thromboprophylaxis is not only to prevent PE but also to prevent symptomatic venous thromboembolisms, including deep venous thrombosis, pulmonary embolisms, and VTE-related deaths. Only patients who underwent Grade IV surgery were enrolled. The results of this study could not be extrapolated to other surgeries. Therefore, it is necessary to carry out further studies to verify these factors through prospective and multicenter studies.

In conclusion, we developed a novel structured approach for selecting risk factors for PE in postoperative patients. This tool provided a consistent, accurate, and efficacious method for risk assessment. This finding may help decision-makers weigh the risk of PE and appropriately select PE prevention strategies.

## Supplementary Information

Supplementary Information.
