# Supplementary material for: Development and validation of a novel risk assessment model to estimate the probability of pulmonary embolism in postoperative patients
Source: Sci Rep. 2021 Sep 10;11:18087. doi: 10.1038/s41598-021-97638-0 (PMC8433319; doi:10.1038/s41598-021-97638-0)
Supplement: Supplementary file 1 — Supplementary Information. [file 41598_2021_97638_MOESM1_ESM.docx]

Appendix 1：Show 25926 patients with 53 clinical and laboratory variables

| **Variables** | **Class** | **Overall(n=25926)** | **Training set (n=15922)** | **Testing set (n=10004)** | **P value** |
| --- | --- | --- | --- | --- | --- |
| Age |  | 55.00 [45.00, 67.00] | 55.00 [45.00, 67.00] | 55.00 [44.00, 67.00] | 0.553 |
| Sex | Female | 14625 (56.4) | 8959 (56.3) | 5666 (56.6) | 0.568 |
| ABO type | O | 9218 (35.6) | 5624 (35.3) | 3594 (35.9) | 0.676 |
|  | AB | 2267 (8.7) | 1413 (8.9) | 854 (8.5) |  |
|  | A | 7795 (30.1) | 4792 (30.1) | 3003 (30.0) |  |
|  | B | 6646 (25.6) | 4093 (25.7) | 2553 (25.5) |  |
| Rh type | negative | 99 (0.4) | 57 (0.4) | 42 (0.4) | 0.495 |
| Smoking | Y | 5639 (21.8) | 3442 (21.6) | 2197 (22.0) | 0.524 |
| Drinking | Y | 6363 (24.5) | 3898 (24.5) | 2465 (24.6) | 0.784 |
| Preoperative venous thrombosis of lower extremity | Y | 220 (0.8) | 137 (0.9) | 83 (0.8) | 0.847 |
| Anesthesia mode | general anesthesia | 1053 (4.1) | 651 (4.1) | 402 (4.0) | 0.805 |
| Hypertension | Y | 6985 (26.9) | 4327 (27.2) | 2658 (26.6) | 0.29 |
| Diabetes | Y | 1949 (7.5) | 1204 (7.6) | 745 (7.4) | 0.751 |
| Coronary heart disease | Y | 1342 (5.2) | 826 (5.2) | 516 (5.2) | 0.939 |
| Malignant tumor | Y | 7058 (27.2) | 4382 (27.5) | 2676 (26.7) | 0.178 |
| COPD | Y | 295 (1.1) | 176 (1.1) | 119 (1.2) | 0.574 |
| Superficial phlebitis | Y | 1 (0.0) | 1 (0.0) | 0 (0.0) | 1 |
| Paralysis | Y | 11 (0.0) | 6 (0.0) | 5 (0.0) | 0.874 |
| Stroke | Y | 553 (2.1) | 348 (2.2) | 205 (2.0) | 0.486 |
| Anticoagulants were used 3 days after operation | Y | 13619 (52.5) | 8405 (52.8) | 5214 (52.1) | 0.299 |
| Heparin was used within 3 days after operation | Y | 13330 (51.4) | 8222 (51.6) | 5108 (51.1) | 0.37 |
| Admission systolic blood pressure |  | 129.00 [116.00, 144.00] | 129.00 [116.00, 144.00] | 129.00 [116.00, 144.00] | 0.945 |
| Admission diastolic pressure |  | 77.00 [69.00, 86.00] | 77.32 [69.00, 86.00] | 77.00 [70.00, 86.00] | 0.602 |
| Admission heart rate |  | 78.00 [71.00, 88.00] | 78.00 [71.00, 88.00] | 78.00 [70.00, 88.00] | 0.158 |
| Admission temperature |  | 36.80 [36.50, 37.10] | 36.80 [36.50, 37.10] | 36.80 [36.50, 37.10] | 0.741 |
| White blood cell count |  | 6.23 [5.02, 7.95] | 6.23 [5.01, 7.96] | 6.22 [5.03, 7.94] | 0.87 |
| Lymphocyte percentage |  | 0.27 [0.19, 0.34] | 0.27 [0.19, 0.34] | 0.27 [0.19, 0.34] | 0.678 |
| neutrophils percentage -last |  | 0.64 [0.57, 0.73] | 0.64 [0.57, 0.73] | 0.64 [0.57, 0.73] | 0.842 |
| neutrophils percentage -max |  | 6.62 [5.83, 7.63] | 6.62 [5.83, 7.63] | 6.62 [5.82, 7.65] | 0.971 |
| Platelet |  | 225.00 [184.00, 271.00] | 225.00 [184.00, 271.00] | 224.00 [184.00, 271.00] | 0.821 |
| Red blood cell |  | 4.39 [4.03, 4.75] | 4.39 [4.03, 4.74] | 4.39 [4.03, 4.75] | 0.872 |
| Red blood cell distribution width |  | 0.13 [0.12, 0.13] | 0.13 [0.12, 0.13] | 0.13 [0.12, 0.13] | 0.949 |
| hemoglobin |  | 131.00 [119.00, 143.00] | 131.00 [119.00, 142.00] | 131.00 [119.00, 143.00] | 0.872 |
| Fasting blood glucose-last |  | 5.17 [4.76, 5.73] | 5.18 [4.76, 5.74] | 5.16 [4.75, 5.73] | 0.137 |
| Fasting blood glucose-max |  | 5.26 [4.82, 5.91] | 5.27 [4.83, 5.92] | 5.25 [4.81, 5.90] | 0.152 |
| Urea nitrogen-last |  | 5.15 [4.20, 6.30] | 5.15 [4.20, 6.30] | 5.16 [4.19, 6.30] | 0.693 |
| Urea nitrogen-max |  | 5.35 [4.37, 6.59] | 5.36 [4.37, 6.59] | 5.35 [4.35, 6.60] | 0.902 |
| Creatinine |  | 60.00 [51.00, 71.00] | 60.00 [51.00, 71.00] | 60.00 [51.00, 71.00] | 0.92 |
| Albumin |  | 41.60 [38.50, 44.20] | 41.60 [38.50, 44.20] | 41.53 [38.50, 44.10] | 0.389 |
| total bilirubin |  | 12.00 [9.00, 15.90] | 12.00 [9.06, 15.88] | 12.00 [9.00, 16.00] | 0.514 |
| Indirect bilirubin |  | 8.02 [6.00, 10.60] | 8.00 [6.00, 10.50] | 8.10 [6.00, 10.65] | 0.566 |
| Direct bilirubin |  | 3.90 [2.90, 5.40] | 3.90 [2.90, 5.39] | 3.90 [2.90, 5.40] | 0.489 |
| calcium |  | 2.27 [2.19, 2.36] | 2.27 [2.19, 2.36] | 2.27 [2.19, 2.36] | 0.193 |
| magnesium |  | 0.92 [0.87, 0.96] | 0.92 [0.87, 0.96] | 0.92 [0.87, 0.96] | 0.632 |
| creatine kinase |  | 86.00 [60.00, 138.00] | 86.00 [60.00, 137.00] | 86.00 [60.00, 140.00] | 0.807 |
| creatine kinase isoenzymelast |  | 15.00 [12.00, 19.05] | 15.00 [12.00, 19.00] | 15.00 [12.00, 19.35] | 0.155 |
| total cholesterol |  | 4.47 [3.89, 5.07] | 4.47 [3.89, 5.07] | 4.47 [3.87, 5.06] | 0.551 |
| Triglyceride |  | 1.33 [0.97, 1.82] | 1.33 [0.97, 1.82] | 1.33 [0.97, 1.82] | 0.786 |
| HDL |  | 1.17 [0.99, 1.35] | 1.17 [0.99, 1.35] | 1.17 [0.99, 1.35] | 0.968 |
| LDL |  | 2.57 [2.11, 3.08] | 2.57 [2.11, 3.08] | 2.57 [2.10, 3.07] | 0.741 |
| ADA |  | 9.93 [8.00, 12.00] | 9.92 [8.00, 12.00] | 9.93 [8.00, 12.00] | 0.61 |
| PT |  | 13.00 [12.50, 13.60] | 13.00 [12.50, 13.60] | 13.00 [12.50, 13.60] | 0.562 |
| APTT |  | 36.20 [33.70, 38.90] | 36.10 [33.70, 38.90] | 36.20 [33.80, 39.00] | 0.242 |
| TT |  | 16.00 [15.40, 16.50] | 16.00 [15.40, 16.50] | 16.00 [15.40, 16.50] | 0.494 |
| Fibrinogen |  | 3.28 [2.80, 3.90] | 3.28 [2.80, 3.90] | 3.28 [2.80, 3.90] | 0.913 |
| D-dimer |  | 0.79 [0.43, 1.66] | 0.79 [0.43, 1.64] | 0.81 [0.44, 1.68] | 0.115 |
